# Clinical Risk Management and Postoperative Outcomes After Colorectal Resection: A Retrospective Observational Study

**DOI:** 10.3390/jpm16040216

**Published:** 2026-04-15

**Authors:** Laura Ambrosi, Giorgio Ammerata, Maurizio Mastrapasqua, Gianmarco Sirago, Valentina Cianci, Biagio Solarino, Alessandro Dell’Erba, Davide Ferorelli, Michele Simone

**Affiliations:** 1 Section of Legal Medicine, Department of Interdisciplinary Medicine, University of Bari, Piazza Giulio Cesare, 11, 70124 Bari, Italy; lauraambrosi9@gmail.com (L.A.); maurizio.mastrapasqua@uniba.it (M.M.); gianmarco.sirago@uniba.it (G.S.); biagio.solarino@uniba.it (B.S.); alessandro.dellerba@uniba.it (A.D.); davideferorelli@gmail.com (D.F.); 2 Department of General Surgery, “Di Venere” Hospital, Via Ospedale Di Venere, 1, 70131 Bari, Italy; valentina.cianci@asl.bari.it (V.C.); michele.simone@asl.bari.it (M.S.)

**Keywords:** clinical risk management, colorectal surgery, postoperative complications, healthcare, patient safety

## Abstract

**Background**: Postoperative complications after colorectal cancer surgery imply challenges to patient safety, recovery, and healthcare resources. Clinical Risk Management (CRM) is vital for reducing complications. This study aims to provide a comprehensive overview of short-term outcomes in a high-volume hospital over four years, evaluating the impact of complications through the lens of CRM. **Methods**: A retrospective cohort study was conducted on 483 patients (332 colon tumors, 151 rectal tumors) who underwent surgical resection. Data were extracted from the internal database, including demographic characteristics, diagnoses, surgical approaches, types of anastomoses, histological grades, and postoperative outcomes. Complications were categorized using the Clavien–Dindo system (grades I–V). Statistical analyses examined the link between clinical variables and complications. **Results**: Postoperative complications occurred in 44 (9.1%) patients in 483 cases. Among the 44 patients with postoperative complications, the most frequent events were anastomotic leakage (AL) (9/44, 20.5%; 9/483, 1.9% of the total cohort) and postoperative hemorrhage (POH) (8/44, 18.2%; 8/483, 1.7% of the total cohort). Moreover, complications were accompanied by an extended hospital stay and a higher in-hospital mortality (15.9% vs. 0%). The number of recorded postoperative follow-up visits differed significantly across complication severity categories. The overall in-hospital survival rate was 98.6%. **Conclusions**: The low rates of complications and in-hospital mortality observed in this cohort were documented within a hospital operating under a mandatory institutional CRM framework. However, due to the retrospective single-arm design, these findings should be interpreted as descriptive and hypothesis-generating rather than causal. The Clavien–Dindo system provided a useful tool for grading complication severity and supporting postoperative management. These findings support continued refinement of perioperative care pathways and further comparative studies on CRM implementation.

## 1. Introduction

Colorectal Cancer (CRC) remains one of the most prevalent and lethal tumors worldwide. It is the third most common cancer globally, and it is the second leading cause of cancer-related deaths [1]. For example, in Italy, nearly 49,000 new cases were diagnosed in 2024 [2]. Risk factors for CRC include diet, genetic predisposition (e.g., Lynch syndrome, familial adenomatous polyposis), inflammatory bowel disease, and sedentary lifestyle [3]. Despite the advancements in screening programs and treatment therapies, many patients are diagnosed at advanced stages, requiring complex surgical interventions [4].

Surgery remains the cornerstone of curative treatment; however, it is associated with a significant risk of postoperative complications [5,6], ranging from minor infections to life-threatening events such as anastomotic leakage (AL) and sepsis [7,8]. Regarding complications in CRC surgery, the postoperative mortality rate ranges from 2% to 9% [9,10].

In surgical procedures, CRM is a systematic approach to enhance patient safety and quality of care. It is based on the identification, assessment, and mitigation of potential risks [11].

Effective CRM incorporates evidence-based tools and organizational practices, such as preoperative checklists, intraoperative safety protocols, postoperative monitoring systems, M&M meetings, and the ongoing training of clinical staff [12]. These instruments, with a standardized surgical technique, help to reduce adverse events and early and late complications. Adherence to the surgical safety checklist was associated with reduced complications, morbidity, and mortality related to surgery [13].

Several structured hospital-wide CRM programs, such as anonymous incident reporting systems, clinical audits, and team-based risk governance, have already demonstrated measurable decreases in adverse event rates across surgical units. These interventions also foster a culture of patient safety and promote shared accountability among clinical teams.

Numerous studies have assessed the risk of various postoperative complications. These studies aimed to reduce the incidence of recurrent complications by mitigating risk factors during the surgical procedure and in the postoperative period [14,15].

Furthermore, a series of scores has been developed to evaluate preoperative risk and stratify the surgical risk for a specific patient.

For instance, the Charlson Comorbidities Index (CCI) assigns a risk score based on the patient’s comorbidities before the operation; the American Society of Anaesthesiologists score (ASA score), instead, evaluates a purely anesthesiologic risk [16,17]. The CR-POSSUM (Physiological and Operative Severity Score for the enumeration of Mortality) score is another tool used to predict morbidity and post-operative mortality following major colorectal surgery [18,19]. Despite these scores, the accuracy of predicting perioperative complications remains a topic of debate.

Within the broader framework of personalized medicine, colorectal surgery increasingly requires risk-adapted perioperative management rather than a uniform postoperative pathway. Patients differ in age, comorbidity burden, tumor site, surgical complexity, and expected recovery trajectories; therefore, complication prevention, monitoring intensity, and escalation strategies should be tailored to individual risk. In this context, CRM may provide the organizational tools needed to translate perioperative risk stratification into more personalized postoperative care.

The Clavien–Dindo classification is a widely standardized method for evaluating the quality and safety of surgical care; it categorizes postoperative complications by severity and the type of treatment required [20].

In this scenario, CRM becomes an essential step in oncological colorectal surgery. Patients who undergo complex surgeries are often older and have several comorbidities. Indeed, surgical complications increase morbidity and mortality, have a dramatic effect on prolonged hospital stays, hinder long-term functional recovery, and strain healthcare resources. CRM in surgical oncology focuses on reducing care variability and enhancing intra-team decision-making by adopting structured, guideline-based workflows and predictive tools.

This study investigates the role of CRM in addressing postoperative complications following CRC surgery. The aim of the current study, which analyses a cohort of surgical cases over four years, is to identify patterns in outcomes, determine predictors of adverse events, and evaluate the clinical outcomes observed within a mandatory institutional CRM framework. Ultimately, the findings may inform the refinement of surgical protocols, provide further support for the development of targeted CRM interventions, and contribute to more personalized, risk-stratified postoperative care.

## 2. Materials and Methods

This retrospective, observational study included 483 surgical procedures for colorectal cancer, suspected or confirmed, over four years (2019–2023); 332 patients underwent resection for colonic lesions and 151 for rectal lesions within the colorectal oncologic pathway. Histology-negative cases were retained in the cohort because these patients underwent resection within the same colorectal oncologic pathway for lesions strongly suspicious for malignancy or for large polyps not amenable to endoscopic removal. For each patient, we extracted: age (>18 years), sex (male/female), tumor location (right/left colon or rectum), type of surgery (laparotomy or laparoscopy), and type of anastomosis performed. Emergency procedures, non-oncological surgeries, and incomplete data sets were considered as exclusion criteria. Ethical approval is not required due to the policy of Di Venere Hospital’s Ethics Committee for a retrospective study characterized by a surgical procedure. All surgical procedures were performed by the same senior surgeon (M.S.) using standardized institutional techniques; each patient signed an informed consent form before the procedure.

Our department, as the entire hospital, implements a mandatory CRM protocol for all major surgical procedures. This framework is integrated into the preoperative, intraoperative, and postoperative phases. Preoperatively, the WHO Surgical Safety Checklist (Sign-in phase) is mandatory for patient identification and site verification. Intraoperatively, standardization is ensured through a single-surgeon approach and the completion of the Time-out and Sign-out sections of the checklist. Postoperatively, all adverse events are systematically reviewed during monthly multidisciplinary Morbidity and Mortality (M&M) meetings. While individual checklist compliance rates were not extracted as a primary study variable, these procedures represent the institutional standard of care and were applied to the entire cohort.

After surgery, anatomopathologists examined the surgical specimen. Postoperative follow-up visits were documented in the institutional records for most patients, with 78.7% undergoing between 1 and 5 recorded follow-up assessments. These visits refer to recorded postoperative surgical follow-up assessments within the institutional pathway and do not include oncology appointments related to adjuvant chemotherapy or radiotherapy. Therefore, this variable was interpreted as a descriptive indicator of postoperative monitoring rather than as a baseline predictor.

Postoperative complications were identified through review of the internal surgical database, inpatient clinical records, discharge documentation, and scheduled postoperative surgical follow-up visits. In addition, adverse events were systematically discussed during monthly multidisciplinary M&M meetings within the institutional CRM framework. Complications were recorded according to the Clavien–Dindo classification. Because this was a retrospective study based on institutional records, complications occurring after discharge and managed outside our hospital may not have been fully captured.

Complications were categorized using the Clavien–Dindo classification: Grade I (minor deviation from normal recovery), Grade II (requiring pharmacological treatment), Grade III (requiring surgical, endoscopic, or radiological intervention), Grade IV (life-threatening complications requiring ICU care), and Grade V (death). Beyond AL and POH, several complications were identified: abscess, fistula, occlusion, sepsis, and intensive care unit recovery.

### Statistical Analysis

Descriptive statistics were used to summarize demographic and clinical characteristics. Continuous variables were expressed as mean ± standard deviation or median and interquartile range, according to data distribution, whereas categorical variables were reported as counts and percentages. Univariable associations with postoperative complications were assessed using Student’s *t*-test or the Mann–Whitney U test for continuous variables and the chi-square test or Fisher’s exact test for categorical variables, as appropriate.

To identify factors independently associated with overall postoperative complications, a multivariable logistic regression model was fitted with postoperative complication status (yes/no) as the dependent variable. Covariates were selected a priori on clinical grounds and included age, sex, tumor location, surgical approach, and final histology. To reduce model overfitting, age was analyzed as a continuous variable per 10-year increase, tumor location was dichotomized as colon versus rectum, and histology was dichotomized as malignant versus non-malignant. Adjusted odds ratios (aORs) with 95% confidence intervals (95% CIs) were reported.

Leakage-related events were explored separately in the subgroup of patients who underwent an anastomosis. Because only nine such events were recorded, the adjusted analysis was considered exploratory and interpreted with caution. The same covariates were entered into this model.

Variables occurring after surgery and potentially lying on the causal pathway of complications, such as length of stay, number of postoperative follow-up visits, treatment of complications, Clavien–Dindo grade, and mortality, were not included as predictors in multivariable models. A two-sided *p*-value < 0.05 was considered statistically significant.

## 3. Results

A total of 483 patients were included in the study; the average age was 68.8 ± 11.3 years, with a slight male predominance (54.7%). In total, 68.7% of patients belonged to the colonic group, while 31.3% belonged to the rectal group. The laparoscopic approach was the most utilized (70.3%); in contrast, the open approach was used in fewer than 30% of cases. Histopathological grading revealed that 46.2% of cases were classified as GII (moderate differentiation), while GIII (poorly differentiated) and GI (well differentiated) accounted for 21.4% and 18.1%, respectively; only 14.3% had no malignancy. In fact, 14.3% of patients had large polyps (over 3 cm) or suspected lesions that were not endoscopically removed (Table 1). Histopathological examination showed that 68 cases (14.3%) were negative for malignancy. These patients had nonetheless undergone surgery because of lesions considered highly suspicious for CRC or because of large polyps not suitable for endoscopic removal.

Regarding complications, they were classified as grade II (43.2%) and grade III (29.5%). Postoperative complications occurred in 44 patients (9.1%). Anastomotic leakage occurred in 9/483 patients (1.9% of the total cohort), corresponding to 20.5% of all recorded complications, whereas postoperative hemorrhage occurred in 8/483 patients (1.7% of the total cohort), corresponding to 18.2% of complications.

The mean hospital stay length was 12.6 ± 5.8 days. A substantial proportion of patients (78.7%) had between 1 and 5 recorded postoperative follow-up visits. The overall recovery rate was 98.6%, while the in-hospital mortality rate was 1.4%.

The comparison between the colonic and rectal groups is summarized in Table 2. Significant between-group differences were observed in the frequency of anastomosis (93.7% vs. 76.8%; *p* < 0.0001), in the distribution of postoperative follow-up visits (*p* = 0.0267), and in the treatment of complications (*p* = 0.0469). In particular, patients in the colonic group more frequently underwent 1–5 recorded postoperative follow-up visits (81.3% vs. 72.8%), whereas patients in the rectal group more often had 6–10 follow-up visits (11.9% vs. 4.5%). Among patients with complications, colonic cases were more often managed with reoperation (46.2%), whereas rectal cases were more frequently treated with pharmacological therapy and/or transfusion (61.1%).

**Figure 1:**
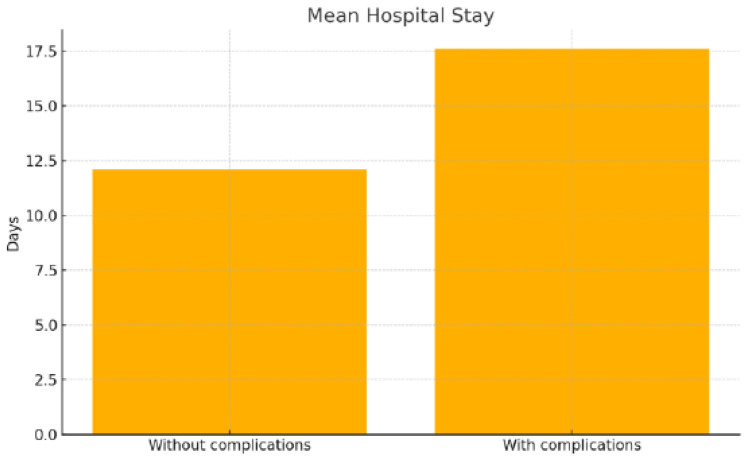
Mean duration of hospital stay in patients with and without complications.

**Figure 2:**
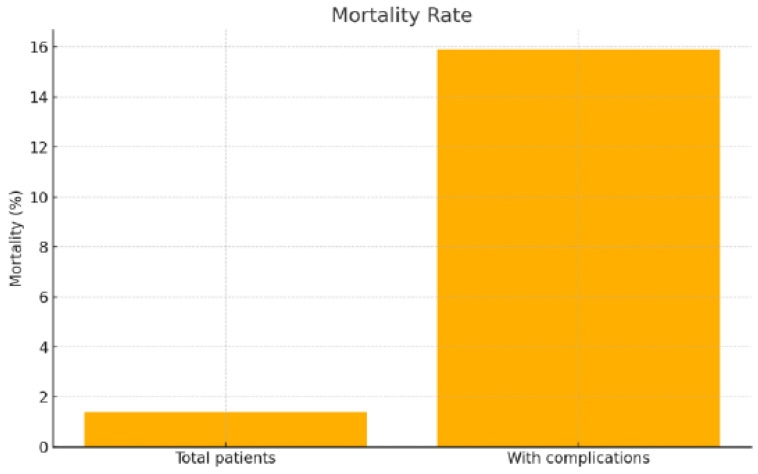
Comparison of mortality rates in the total cohort and among patients with complications.

Patients with postoperative complications had a significantly longer hospital stay (17.6 ± 10.0 vs. 12.1 ± 4.9 days; *p* < 0.0001), a higher rate of no follow-up (36.4% vs. 10.7%; *p* < 0.0001), and a significantly worse outcome (mortality of 15.9% vs. 0%; *p* < 0.0001) (Figure 1 and Figure 2).

The comparison of demographic, clinical, and surgical variables between patients with and without postoperative complications are summarized in Table 3. The distribution of complication types according to Clavien–Dindo grade and the clinical characteristics of patients across complication severity categories are summarized in Table 4 and Table 5. Anastomotic leakage was most frequently observed among grade III complications, although cases were also recorded in grades I and V. Hemorrhage was distributed across grades I–III, whereas fistula cases were distributed across grades I–III. Grade III complications were managed predominantly by reoperation, while grade II complications were treated mainly with pharmacological therapy and/or transfusion.

In multivariable logistic regression analysis, none of the predefined baseline variables retained an independent association with overall postoperative complications after adjustment for age, sex, tumor location, surgical approach, and final histology. Specifically, age (aOR 1.18, 95% CI 0.88–1.59; *p* = 0.267), male sex (aOR 1.33, 95% CI 0.70–2.52; *p* = 0.382), rectal versus colonic location (aOR 1.63, 95% CI 0.86–3.10; *p* = 0.134), open versus laparoscopic approach (aOR 1.34, 95% CI 0.68–2.64; *p* = 0.403), and non-malignant versus malignant histology (aOR 0.75, 95% CI 0.28–2.00; *p* = 0.572) were not significant independent predictors.

Leakage-related events were explored separately in the subgroup of patients who underwent anastomosis. Because only nine events were recorded, this adjusted model was interpreted as exploratory. In this analysis, none of the included baseline covariates showed a significant independent association with AL. These findings suggest that the crude associations observed in univariable analyses may have been influenced by case-mix and perioperative complexity rather than by single independent baseline predictors.

## 4. Discussion

This observational and retrospective study provides a comprehensive analysis of postoperative outcomes in a large cohort of patients undergoing colorectal surgery. Special attention was given to the incidence, grading, and management of complications. The overall complication rate was 9.1%, which is lower than the rates commonly reported in the international literature, ranging from 20% to 49% [21,22]. However, given the retrospective observational design and the absence of a comparator group, these findings should be interpreted as descriptive outcomes observed within our institutional pathway rather than as evidence of the causal effectiveness of any single protocol. Differences from published complication rates may also reflect variations in case-mix, definitions, and ascertainment of postoperative events, particularly those occurring after discharge.

The relatively low complication rate observed in our cohort should also be interpreted in light of possible ascertainment bias, as retrospective institutional data may underestimate complications diagnosed after discharge or managed in other settings. At the same time, our findings indicate that conducting the procedures within a highly standardized operative approach, led by a consistent senior surgeon (M.S.), may have contributed to procedural uniformity and the observed outcomes, including an anastomotic leak rate of 1.9%. While a single-operator setting strengthens internal consistency by reducing inter-operator variability, it also limits reproducibility across different teams, thereby reducing the external validity and generalizability of the results. This value was very low compared with the incidence currently reported in colorectal surgery, which ranges from 2.8% to 30%, with up to 75% of cases occurring in rectal anastomosis [23,24,25,26]. Another encouraging finding is the mortality rate of only 1.4%. While we did not capture case-level CRM fidelity metrics, such as individual checklist completion rates, and therefore cannot infer a direct causal link, our results compare favorably with outcomes reported in standardized, high-reliability perioperative settings. These elements describe how integrating institutional CRM with a strict, standardized approach may yield satisfactory outcomes for oncological patients.

These outcomes were observed in a setting characterized by a standardized operative approach and an established institutional CRM framework. The implementation of perioperative safety tools, such as surgical safety checklists and structured team training, is supported by a substantial body of literature reporting improvements in teamwork and communication, as well as associations with reduced surgical morbidity and mortality [27,28,29]. Conceptually, these interventions reflect a systems approach to safety, shifting attention from individual error to the strengthening of organizational barriers and adaptive capacity [30].

These findings should also be interpreted in light of some important methodological limitations, which are discussed separately below.

Complications such as AL, POH, and sepsis were among the most frequently observed and carried several clinical consequences, including prolonged hospital stay and increased mortality. These findings underscore the need for robust CRM systems to proactively mitigate such risks. Indeed, CRM involves the early recognition of adverse events and the implementation of standardized surveillance protocols, while escalation of care remains an essential component of a safe surgical environment. The Clavien–Dindo classification proved to be a practical framework for assessing the severity of complications and guiding clinical decision-making. Higher-grade complications were significantly associated with more intensive therapies, and the distribution of complications by grade further underscores the importance of personalized postoperative planning and monitoring.

Law et al. also reported that medical complications, such as sepsis, are more common in patients with colon cancer, whereas surgical complications, such as AL or POH, are more common in patients with rectal cancer [31]. In our cohort, a minimally invasive surgical approach, such as laparoscopy, was associated with slightly lower complication rates, suggesting advantages in reduced tissue trauma, faster recovery, and lower infection risk. It has been widely demonstrated that laparoscopy can reduce surgical stress responses, improve postoperative outcomes, and decrease postoperative morbidity and mortality [32,33,34]. According to Curtis et al., laparoscopy appears safer in oncological surgery; moreover, when combined with an ERAS protocol, the laparoscopic approach correlates with greater long-term survival [35]. Tailored postoperative strategies for oncological patients could therefore improve CRM by concentrating resources, especially on higher-risk patients.

From a systemic perspective, the analysis of surgical codes and their correlation with specific complication types suggests that certain procedures have intrinsically higher risk profiles. These findings, when combined with surgical safety checklists, intraoperative briefings, and M&M audits, could lead to improvements in clinical pathways. Risk management strategies such as Enhanced Recovery After Surgery (ERAS) protocols, perioperative antibiotic stewardship, adherence to sterile techniques, and multidisciplinary decision-making pathways are crucial for reducing variability and enhancing surgical outcomes. The implementation of such routines is essential for standardizing care and minimizing the risk of preventable complications.

A root cause analysis is useful for identifying both remote and immediate causes of safety incidents; however, it does not focus on implementing effective measures to prevent their recurrence [36]. In contrast, the clinical audit is a tool aimed at preventing new events and is a widely standardized and validated CRM instrument. The audit process facilitates oversight of internal operations and verifies their alignment with quality standards and guidelines [37]. Several studies have demonstrated that periodic clinical audits are associated with improved quality of care in colorectal surgery [38]. In our setting, the combination of institutional CRM practices, perioperative standardization, and structured postoperative surveillance was associated with low observed rates of complications and in-hospital mortality; however, no causal inference can be made from the present study design.

The inclusion of histology-negative resections may have introduced some heterogeneity, since these patients may differ from those with histologically confirmed malignancy in terms of biological behavior and postoperative risk profile. However, these procedures were performed within the same oncologic surgical pathway and therefore reflect a real-world surgical indication scenario. Moreover, Electronic Health Records (EHRs) can play a crucial role in enhancing patient safety, although they were not used in this study. EHRs facilitate communication among healthcare providers, simplify patient history tracking, and may decrease medical errors [39,40]. Their advantages include improved continuity of care, greater availability and accessibility of data, and enhanced support for clinical decision-making, despite potential drawbacks such as confidentiality and data security concerns, staff resistance, and interoperability challenges [41,42]. Their implementation across healthcare settings remains an important goal.

In conclusion, the present findings support the relevance of maintaining structured CRM processes within colorectal oncologic surgery, while acknowledging that the present study was not designed to isolate the independent effect of CRM on outcomes. By identifying modifiable risk factors, promoting adherence to follow-up care, and optimizing perioperative strategies, healthcare institutions can enhance patient outcomes and safety while minimizing the impact of complications. Integrating predictive analytics and artificial intelligence into real-time decision support systems may further enhance the ability to anticipate and mitigate surgical risk in the future.

## 5. Limitations

The present study has several limitations. First, its retrospective single-center design makes it vulnerable to incomplete data capture and residual confounding. Second, the absence of a comparator group does not allow for causal inference regarding the independent effect of CRM on outcomes. Third, complications occurring after discharge and managed outside our institution may have been underrepresented. Fourth, the single-surgeon setting, while improving procedural consistency, limits external validity and generalizability. Finally, some clinically relevant oncologic variables, including TNM staging and neoadjuvant treatment data, were not systematically available for all cases.

## 6. Conclusions

CRM is vital for improving safety and outcomes in CRC surgery. This study describes postoperative outcomes observed in the context of a structured, institutional CRM framework, combined with standardized surgical techniques. While the retrospective nature of the study limits causal inferences, the low incidence of complications underscores the value of maintaining high-reliability safety protocols in oncological colorectal surgery. Institutional commitment to training, monitoring, and ongoing protocol improvement is essential.

Utilizing standardized classification systems, such as the Clavien–Dindo system, and routinely employing CRM tools fosters a culture of safety and quality in surgical care. Future efforts should focus on multidisciplinary collaboration, technological integration, and continuous feedback to enhance protocols and reduce preventable harm. In particular, multicenter comparative studies should assess whether structured CRM pathways can support more personalized postoperative surveillance and risk-adapted care after colorectal resection.

## Figures and Tables

**Table 1:** Demographic, Clinical, and Surgical Characteristics of the Study Population.

Variable	Category	Overall
		483
Age		68.8 ± 11.3
Sex	F	219 (45.3)
	M	264 (54.7)
Group	Colon	332 (68.7)
	Rectum	151 (31.3)
Postoperative follow-up visits	0	63 (13.0)
	1–5	380 (78.7)
	6–10	33 (6.8)
	>10	7 (1.4)
Length of stay		12.6 ± 5.8
Outcome	Healing	476 (98.6)
	Death	7 (1.4)
Histology	Negative	68 (14.3)
	G I	86 (18.1)
	G II	220 (46.2)
	G III	102 (21.4)
Type of surgery	Laparotomy	186 (38.5)
	Laparoscopy	297 (61.5)
Complications	No	439 (90.9)
	Yes	44 (9.1)
Type of complications	Abscess	2 (4.5)
	Anastomotic leakage	9 (20.5)
	Haemorrhage	8 (18.2)
	Fistula	4 (9.1)
	ICU	4 (9.1)
	Occlusion	3 (6.8)
	Sepsis	3 (6.8)
	Other	11 (25.0)
Clavien–Dindo	Grade I	5 (11.4)
	Grade II	19 (43.2)
	Grade III	14 (31.8)
	Grade V	6 (13.6)
Treatment	Pharmacological	1 (2.3)
	Pharmacological/transfusion	17 (38.6)
	Dressing/drainage	11 (25.0)
	Reoperation	15 (34.1)

Percentages for “Type of complications”, “Clavien–Dindo”, and “Treatment” are calculated on the subgroup of patients with postoperative complications (*n* = 44), unless otherwise specified.

**Table 2:** Comparison between the colonic and rectal groups.

Variable	Category	Colon (*n* = 332)	Rectum (*n* = 151)	*p* Value
Anastomosis	No	21 (6.3)	35 (23.2)	<0.0001 **
	Yes	311 (93.7)	116 (76.8)	
Postoperative follow-up visits	0	42 (12.7)	21 (13.9)	0.0267 **
	1–5	270 (81.3)	110 (72.8)	
	6–10	15 (4.5)	18 (11.9)	
	>10	5 (1.5)	2 (1.3)	
Treatment of complications *	Pharmacological	1 (3.8)	0 (0.0)	0.0469 **
	Pharmacological/transfusion	6 (23.1)	11 (61.1)	
	Dressing/drainage	7 (26.9)	4 (22.2)	
	Reoperation	12 (46.2)	3 (16.7)	

* Percentages for treatment of complications are calculated on the subgroup of patients with recorded postoperative complications: colon *n* = 26; rectum *n* = 18. ** Fisher test.

**Table 3:** Comparison of demographic, clinical, and surgical variables between patients with and without postoperative complications. Continuous variables are expressed as mean ± SD; categorical variables as counts and percentages. *p*-values from Fisher’s exact test here.

Variable	Category	Complications		*p* Value
		NO	YES	
N		439	44	
Age		68.6 ± 11.3	70.8 ± 10.6	0.2128
Sex	F	202 (46.0)	17 (38.6)	0.3487
	M	237 (54.0)	27 (61.4)	
Anastomosis	No	51 (11.6)	5 (11.4)	0.96
	Yes	388 (88.4)	39 (88.6)	
Postoperative follow-up visits	0	47 (10.7)	16 (36.4)	<0.0001 *
	1–5	357 (81.3)	23 (52.3)	
	6–10	29 (6.6)	4 (9.1)	
	>10	6 (1.4)	1 (2.3)	
Length of stay		12.1 ± 4.9	17.6 ± 10.0	<0.0001
Outcome	Healing	439 (100.0)	37 (84.1)	<0.0001 *
	Death	0 (0.0)	7 (15.9)	
Group	Colon	306 (69.7)	26 (59.1)	0.1477
	Rectum	133 (30.3)	18 (40.9)	
Histology	Negative	63 (14.6)	5 (11.4)	0.6354
	G I	78 (18.1)	8 (18.2)	
	G II	196 (45.4)	24 (54.5)	
	G III	95 (22.0)	7 (15.9)	
Type of surgery	Laparotomy	172 (39.2)	30 (68.2)	0.3387
	Laparoscopy	267 (60.8)	14 (31.8)	

* Fisher test.

**Table 4:** Distribution of complication types according to Clavien–Dindo grade and management strategy.

	Abscess (*n* = 2)	Anastomotic Leakage (*n* = 9)	Hemorrhage (*n* = 8)	Fistula (*n* = 4)	ICU (*n* = 4)	Occlusion (*n* = 3)	Sepsis (*n* = 3)	Other (*n* = 11)
**Clavien–Dindo grade**								
Grade I	0 (0.0)	1 (11.1)	1 (12.5)	1 (25.0)	1 (25.0)	0 (0.0)	0 (0.0)	1 (9.1)
Grade II	1 (50.0)	0 (0.0)	4 (50.0)	1 (25.0)	3 (75.0)	2 (66.7)	1 (33.3)	7 (63.6)
Grade III	1 (50.0)	6 (66.7)	3 (37.5)	2 (50.0)	0 (0.0)	1 (33.3)	1 (33.3)	0 (0.0)
Grade V	0 (0.0)	2 (22.2)	0 (0.0)	0 (0.0)	0 (0.0)	0 (0.0)	1 (33.3)	3 (27.3)
**Management strategy**								
Pharmacological	0 (0.0)	0 (0.0)	0 (0.0)	0 (0.0)	0 (0.0)	0 (0.0)	0 (0.0)	1 (9.1)
Pharmacological/transfusion	1 (50.0)	0 (0.0)	3 (37.5)	0 (0.0)	2 (50.0)	2 (66.7)	2 (66.7)	7 (63.6)
Dressing/drainage	0 (0.0)	1 (11.1)	2 (25.0)	3 (75.0)	2 (50.0)	0 (0.0)	0 (0.0)	3 (27.3)
Reoperation	1 (50.0)	8 (88.9)	3 (37.5)	1 (25.0)	0 (0.0)	1 (33.3)	1 (33.3)	0 (0.0)

**Table 5:** Clinical, demographic, and surgical characteristics of patients based on complication severity (Clavien–Dindo classification). Continuous variables are shown as mean ± standard deviation, while categorical variables are reported as counts and percentages. * *p*-values are calculated using Fisher’s exact test when appropriate.

Variable	Category	Grade I	Grade II	Grade III	Grade V	*p* Value
N		5	19	14	6	
Age		67.2 ± 15.8	70.4 ± 9.7	70.6 ± 10.2	75.8 ± 10.8	0.5975
Sex	F	3 (60.0)	8 (42.1)	4 (28.6)	2 (33.3)	0.6504 *
	M	2 (40.0)	11 (57.9)	10 (71.4)	4 (66.7)	
Anastomosis	No	1 (20.0)	1 (5.3)	0 (0.0)	3 (50.0)	0.01 *
	Yes	4 (80.0)	18 (94.7)	14 (100.0)	3 (50.0)	
Postoperative follow-up visits	0	2 (40.0)	6 (31.6)	2 (14.3)	6 (100.0)	0.0181 *
	1–5	3 (60.0)	11 (57.9)	9 (64.3)	0 (0.0)	
	6–10	0 (0.0)	1 (5.3)	3 (21.4)	0 (0.0)	
	>10	0 (0.0)	1 (5.3)	0 (0.0)	0 (0.0)	
Length of stay		14.2 ± 5.0	16.9 ± 9.6	20.4 ± 9.1	16.0 ± 16.2	0.6016
Outcome	Healing	5 (100.0)	19 (100.0)	13 (92.9)	0 (0.0)	<0.0001 *
	Death	0 (0.0)	0 (0.0)	1 (7.1)	6 (100.0)	
Group	Colon	4 (80.0)	9 (47.4)	10 (71.4)	3 (50.0)	0.4079 *
	Rectum	1 (20.0)	10 (52.6)	4 (28.6)	3 (50.0)	
Histology	Negative	2 (40.0)	2 (10.5)	1 (7.1)	0 (0.0)	0.4224 *
	G I	1 (20.0)	2 (10.5)	2 (14.3)	3 (50.0)	
	G II	2 (40.0)	11 (57.9)	8 (57.1)	3 (50.0)	
	G III	0 (0.0)	4 (21.1)	3 (21.4)	0 (0.0)	
Type of surgery	Laparotomy	5 (100.0)	13 (68.4)	8 (57.1)	4 (66.7)	0.4531 *
	Laparoscopy	0 (0.0)	6 (31.6)	6 (42.9)	2 (33.3)	
Treatment	Pharmacological	0 (0.0)	1 (5.3)	0 (0.0)	0 (0.0)	<0.0001 *
	Pharmacological/transfusion	0 (0.0)	13 (68.4)	0 (0.0)	4 (66.7)	
	Dressing/drainage	5 (100.0)	5 (26.3)	1 (7.1)	0 (0.0)	
	Reoperation	0 (0.0)	0 (0.0)	13 (92.9)	2 (33.3)	

## Data Availability

The original contributions presented in this study are included in the article. Further inquiries can be directed to the corresponding author.
